# The in vivo regulation of heart rate in the murine sinoatrial node by stimulatory and inhibitory heterotrimeric G proteins

**DOI:** 10.1152/ajpregu.00037.2013

**Published:** 2013-05-22

**Authors:** Sonia Sebastian, Richard Ang, Joel Abramowitz, Lee S. Weinstein, Min Chen, Andreas Ludwig, Lutz Birnbaumer, Andrew Tinker

**Affiliations:** ^1^William Harvey Heart Centre, Barts and The London School of Medicine and Dentistry, London, United Kingdom;; ^2^Division of Intramural Research, National Institute of Environmental Health Sciences, Research Triangle Park, North Carolina;; ^3^Metabolic Diseases Branch, National Institute of Diabetes and Digestive and Kidney Diseases/National Institutes of Health, Bethesda, Maryland; and; ^4^Institut für Experimentelle und Klinische Pharmakologie und Toxikologie, Universitaet Erlangen-Nuernberg, Erlangen, Germany

**Keywords:** G protein, heart rate, sinoatrial node

## Abstract

Reciprocal physiological modulation of heart rate is controlled by the sympathetic and parasympathetic systems acting on the sinoatrial (SA) node. However, there is little direct in vivo work examining the role of stimulatory and inhibitory G protein signaling in the SA node. Thus, we designed a study to examine the role of the stimulatory (Gαs) and inhibitory G protein (Gαi2) in in vivo heart rate regulation in the SA node in the mouse. We studied mice with conditional deletion of Gαs and Gαi2 in the conduction system using cre-loxP technology. We crossed mice in which cre recombinase expression was driven by a tamoxifen-inducible conduction system-specific construct with “Gαs floxed” and “Gαi2 floxed” mice. We studied the heart rate responses of adult mice compared with littermate controls by using radiotelemetry before and after administration of tamoxifen. The mice with conditional deletion of Gαs and Gαi2 had a loss of diurnal variation and were bradycardic or tachycardic, respectively, in the daytime. In mice with conditional deletion of Gαs, there was a selective loss of low-frequency power, while with deletion of Gαi2, there was a loss of high-frequency power in power spectral analysis of heart rate variability. There was no evidence of pathological arrhythmia. Pharmacological modulation of heart rate by isoprenaline was impaired in the Gαs mice, but a muscarinic agonist was still able to slow the heart rate in Gαi2 mice. We conclude that Gαs- and Gαi2-mediated signaling in the sinoatrial node is important in the reciprocal regulation of heart rate through the autonomic nervous system.

cardiac automaticity is modulated by both sympathetic and parasympathetic systems, and they are opposing in nature. Noradrenaline from sympathetic nerve efferents and adrenaline released into the circulation from the adrenal medulla accelerate heart rate, while ACh from vagal nerve efferents slows it down. The neurotransmitters and hormones act on the sinoatrial (SA) node to modulate an intrinsic pacemaking clock, the nature of which is controversial. The two main ideas are cycles of membrane pacemaker depolarization generated by hyperpolarization-activated cyclic nucleotide-gated cation channels (also known as *I*_f_ or *I*_h_) and/or the cyclical release of Ca^2+^ from intracellular stores via ryanodine receptors increasing inward sodium-calcium exchanger currents during the diastolic depolarization (3, 6, 11, 13). Noradrenaline and adrenaline bind to β_1_- and β_2_-adrenoreceptors and activate the stimulatory G protein (Gαs). Activated Gαs stimulates adenylyl cyclase, leading to an increase in cAMP level, which directly activates *I*_f_. This also leads to increased activity of PKA, resulting in modulation of downstream effectors, such as intracellular Ca^2+^-handling pathways, for example, phospholamban and the ryanodine receptor. In contrast, slowing of pacemaker depolarization occurs because of ACh binding to muscarinic M_2_ receptors and the activated Gαi\o antagonizes the above pathways. In addition, the free Gβγ subunit released from Gαi\o directly activates the G protein-gated inwardly rectifying K^+^ channel leading to membrane hyperpolarization. It is generally thought that all of these mechanisms contribute to some extent to modulating heart rate (1). These responses are all predicated on signaling networks initiated via Gαs and Gαi\o, and the evidence of their importance is largely based on ex vivo studies of the sinoatrial node and pacemaker cells (17). Furthermore, in vivo heart rate shows variability not reproduced in such studies, and specific frequency components are attributed to sympathetic and vagally mediated cardiovascular reflexes (22). In addition, the issue is intricate at the molecular level. *Gnas* is a complex gene with the potential for generation of long and extralong isoforms (27). Furthermore, there are four main isoforms of Gαi\o (Gαi1, Gαi2, Gαi3, and Gαo), and these are all widely expressed in most tissues with significant possibilities for redundancy (5). We have previously shown that mice with global genetic deletion of Gαi2 have abnormalities of heart rate regulation compatible with a role for that protein in the SA node, and thus, we focus here on that isoform (29). We have not previously investigated the contribution that Gαs makes to heart rate regulation, and this would not be possible in a strictly analogous fashion to our studies on the inhibitory G proteins as global genetic deletion of Gαs is embryonically lethal (27). Thus, we refined our strategy and sought to investigate the significance of Gαs and Gαi2 for physiological heart rate regulation in the sinoatrial node of the adult mouse using a conditional gene-targeting approach.

## MATERIALS AND METHODS

### 

#### Murine husbandry.

Mice were maintained in an animal core facility under U.K. Home Office guidelines relating to animal welfare. All maintenance, breeding, and procedures were covered by project license PPL 70\6732. All mice were kept in a temperature-controlled environment (21–23°C) with 12:12-h dark-light cycles. Animals were allowed access to standard rodent chow and water ad libitum. Mice were studied between 8 and 12 wk of age.

#### Mouse strains and breeding and genotyping.

We interbred several mouse lines. We used mice in which the tamoxifen-inducible CreER^T2^ construct was “knocked” into the pacemaker channel HCN4 locus (HCN4-KiT, referred to as cre+ here), allowing for selective expression of cre recombinase in the cardiac conduction system following intraperitoneal administration of tamoxifen (12). We interbred these with “Gαs floxed” (Gαs^flx\flx^) and “Gαi2 floxed” (Gαi2^flx\flx^) mice. Gαs^flx\flx^ mice have loxP sites placed in introns upstream and downstream of exon 1. Gαi2^flx\flx^ mice have loxP sites placed in introns upstream of exon 2 and downstream of exon 4, and, thus, cre-mediated excision deletes exons 2, 3, and 4, which is equivalent to amino acids 40–155. A description of the targeting strategy to produce both lines of *flx/flx* mice has previously been published (8, 26). After successive rounds of breeding, we generated Gαs^flx\flx^ cre+ mice, Gαi2^flx\flx^ cre+ mice, and littermate “controls”, which consisted of wild-type, cre+ only, and Gαs^flx\flx^ or Gαi2^flx\flx^ only genotypes. We observed no prominent differences among the latter groups and thus pooled them into single littermate control populations. Mice of both sexes were used in this study. The cre recombinase-expressing line is on a C57/BL6 background, Gαs^flx\flx^ is on a C57/BL6 background, and the Gαi2^flx\flx^ is on the 129/Sv background. Tamoxifen (Sigma) was freshly dissolved in sunflower oil, and 1 mg tamoxifen/25 g body wt ip was injected on five consecutive days. Mice were studied 10 days after the last dose.

Mice were tail clipped (∼2 mm) at 3–4 wk of age. Genomic DNA was isolated from tail clips. PCR-based genotyping was then performed on isolated DNA to confirm genotype of individual mice. One-hundred-and-fifty microliters of tail lysis buffer [3.35 ml 2 M Tris·HCl pH 8.8, 1.66 ml 1 M (NH_4_)_2_SO_4_, 1.34 ml 0.5 M MgCl_2_, 0.5 ml Triton X-100, 92.2 ml H_2_O, and 1 ml β-mercaptoethanol] was added, and the samples were heated to 100°C for 10 min in a heating block to denature mouse proteins. Samples were then cooled, and 5 μl of proteinase K (20 mg/ml) was added at 55°C for 12 h. Samples were then reheated to 100°C for a further 10 min and then spun at 13,000 rpm for 30 s in a tabletop centrifuge to sediment tail debris. One microliter of sample (mouse genomic DNA) was used per genotyping PCR. Identification of cardiac *Cre*^**^ expression was determined using the following primer sets: HCN4Ki F5′-CCCGCGCTGGAGTTTCAATA-3′ and HCN4Ki R5′-CTTCGCCCAGTT GATCATGTG-3′. The presence of the *Cre* transgene was determined by the presence or absence of a *Cre* band (383bp). Gαs lox-P (floxed allele) genotyping was performed using the following primers: Gαs LoxF 5′-TTCGG TCTCG TCCCC TTAGT TG-3′ and Gαs LoxR 5′-AACAA ATCGC ACACC CCAGT GAGG-3′ using betaine (1.5 M) in the PCR master mix to obtain a WT band corresponding to ∼216 bp and a Gαs floxed band corresponding to ∼264 bp, respectively. Cre-mediated excision was confirmed by Gαs cre F 5′-GAGAGCGAGAGGAAGACAGC-3′and Gαs cre R 5′-AGCCCTACTCTGTCGCAGTC-3′ primers, which gives a band of ∼250 bp upon cre-mediated excision of exon 1. Gαi2 lox-P (floxed allele) genotyping was performed using the following primers: Gαi2 LoxF 5′-GGA GCC TGG ACT TTG CTT CTG ACC-3′ and Gαi2 LoxR 5′-GGC TAT GAT CCC AAA ACT CCC CG-3′ Gαi2 LoxF2 5′GTG GTA AGC CTG TGT GTT TGT GAG AG-3′. Using Gαi2 LoxF and Gαi2 LoxR as primers and a hotstart PCR strategy, we observed a ∼400-bp band corresponding to WT Gαi2 and a ∼500-bp band corresponding to the Gαi2 lox-P (floxed) allele. Using Gαi2 LoxF2 and Gαi2 LoxR, we observed that a ∼2,200-bp band in the absence of cre-mediated deletion of the “floxed” allele was present while a ∼400-bp band was produced after *Cre-*mediated excision of exons 2 to 4.

#### Real-time RT-PCR on isolated SA nodal tissue.

RNA was isolated from the SA node and ventricle after tamoxifen treatment in both Gαs^flx\flx^ cre+ or Gαi2^flx/flx^ cre+ and control mice using the RNeasy kit (Qiagen, Valencia, CA). cDNA was synthesized using the high-capacity cDNA reverse transcription kit (Applied Biosystems, Foster City, CA). By using TaqMan gene expression assay (Assay ID: Mm03945887_s1), which spans exon 1, Gαs cDNA was quantified. Gαi2 cDNA was then quantified using the TaqMan gene expression assay kit using custom-designed primers spanning exon 2 boundary TGGAGAGTCAGGGAAGAGCA and exon 3 TAGACCACGGCACGGTACT (Applied Biosystems). All genes were assayed in triplicate, and relative gene expression was quantified using the comparative C_T_ method with GAPDH as the reference housekeeping gene (19).

#### Radiotelemetry.

The telemetry probe (TEA-F20; Data Science International, St. Paul, MN) was implanted into mice to record ECG data in the conscious animal, as described previously (29). Briefly, tunnelled electrodes were secured in a lead II configuration connected to a telemetry device, which was implanted intra-abdominally using aseptic surgical technique. After a minimum of a week-long period of surgical recovery, ECG signals were acquired by radio-telemetry. To further examine heart rate modulation in vivo, we used pharmacological provocation with isoprenaline (3.0 μg/kg intraperitoneally) and carbachol (0.5 mg/kg intraperitoneally).

#### Analysis of heart rate.

Initially, mean day and night heart rate (12 h light to dark cycle) were determined over 48 h from heart rate recorded for 15 s every 30 min. Surface ECG parameters, such as PR, QRS, and QT_c_ intervals, were also measured. Subsequently, we acquired R-R interval variability signal from ECG data streamed over 30 min at high sampling frequency (2 kHz), digitized and analyzed using a heart rate variability (HRV) extension module of CHART v7.0 (ADInstruments, Oxford, UK). A threshold-sensing algorithm for R-wave recognition was applied to detect all R-R intervals. Ectopic beats, which occurred only very rarely in this study, were excluded from analysis, and no averaged or interpolated beats were used to replace them. The data set of consecutive R-R intervals (typically, ∼12,000 sinus beats per mouse recording) was then analyzed in both the linear time and frequency domains. Time domain measurements include SD_n-n_, standard deviation of all R-R intervals in sinus rhythm (25), and root mean square SD, as the square root of the mean of the square difference between adjacent normal R-R intervals (9, 18, 25). Frequency domain analysis was performed after fast Fourier transform using 1,024 spectral points and a half overlap within a Welch window. For the frequency domain analysis, cut-off frequencies previously determined to be accurate for mice were used to divide signal into three major components of total power (TP), very low frequency (VLF < 0.4 Hz), low frequency (LF 0.4–1.5 Hz), and high frequency (HF 1.5–4.0 Hz) (9, 10, 28). Normalization to exclude VLF was performed. Normalized low frequency (nLF) = LF/(TP − VLF) × 100 and normalized high frequency (nHF) = HF/(TP − VLF) × 100. The rate-corrected QT interval (QTc) is defined as QTc = QT/sqrt(R − R/100) with R-R measured in milliseconds. Heart rate after pharmacological challenge was determined over a 30-min time course and was measured when it had reached a steady state. The chronotropic effect of isoprenaline and carbachol was expressed as a percentage change of heart rate relative to baseline heart rate prior to drug administration. Intrinsic heart rate was determined in a similar way by administering atropine (1 mg/kg) and propanolol (1 mg/kg) together to achieve autonomic blockade in awake conscious mice with telemetry probes in situ. We also examined the effects of the drugs on HRV by recording 10 min of ECG tracing prior to drug administration and another 10 min after the heart rate had reached a steady state following drug administration.

#### Statistical analysis.

Data are reported as means ± SE. Statistical significance of multiple treatments was determined by one- and two-way ANOVA followed by Dunnett's post hoc analysis, and statistical significance between two groups was determined by two-tailed Student's *t-*tests, as appropriate (GraphPad Prism v4). In all instances, *P* < 0.05 was considered significant.

## RESULTS

### 

#### Generating mice with deletion of Gαs and Gαi2 in the conduction system.

Gαs^flx\flx^ cre+, Gαi2^flx\flx^ cre+ mice and littermate controls were generated as described above. We performed quantitative real-time RT-PCR on RNA extracted from the SA node and ventricle. We normalized measurements in the Gαs^flx\flx^ cre+ and Gαi2^flx\flx^ cre+ mice to expression in control mice (both after the administration of tamoxifen) in ventricle and SA node. There was a significant reduction in relative Gαs and Gαi2 RNA expression in the SA node but not ventricle {Gαs^flx\flx^ cre+ SA node = 0.18 [95% confidence interval (CI) 0.17–0.18], ventricle = 1.01, (95% CI 0.99–1.03); and Gαi2^flx\flx^ cre+ SA node = 0.11 (95% CI 0.09–0.24), ventricle = 1.04 (95% CI 0.80–1.36); *n* = 2 and 3 mice, respectively, in triplicate for all groups}. We also isolated genomic DNA from the tail, cardiac ventricle, and SA node and performed PCR, which confirmed cre-mediated deletion of exon 1 of *Gnas* and exons 2–4 in *Gnai2* in the SA node but not in the ventricle or tail (not shown).

#### Effects on mean heart rate and diurnal variation.

**Fig. 1.**
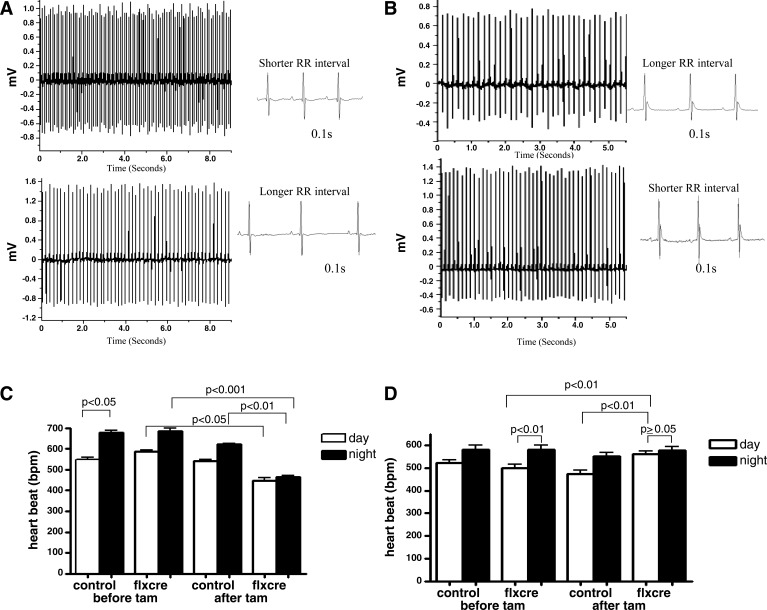
ECG traces and diurnal variation of heart rate. *A* and *C*: representative ECG recordings and mean data for Gα_s_^fl\flx^ cre+ (*n* = 8) and control (*n* = 8). *B* and *D*: for Gαi2^flx\flx^, cre+ (*n* = 12) and control mice (*n* = 9). Values in *B* and *D* are expressed as means ± SE.

Significant diurnal variation in heart rate (HR) was observed in Gαs^flx\flx^ cre+ and Gαi2^flx\flx^ cre+ mice before tamoxifen treatment but not after (Fig. 1). There was a significant decrease in night-time HR in Gαs^flx\flx^ cre+ mice, while there was a significant increase in day-time HR after tamoxifen treatment in Gαi2^flx\flx^ cre+ mice (Fig. 1). In contrast, control mice had preserved diurnal variation, and there was no significant change after tamoxifen treatment (Fig. 1). We examined ECG parameters, and there was no significant difference in PR interval, QRS duration, or any evidence of spontaneous arrhythmia, such as heart block (Fig. 1 and Table 1). However, QTc was shorter in the Gαs^flx\flx^ cre+ mice, but this was not investigated further in this study.

**Table 1.** Summarized ECG and HRV parameters for for Gαs^flx^^\flx^ cre+ (*n* = 8) and control (*n* = 8) and for Gαi2^flx\flx^ cre+ (*n* = 11) and control mice (*n* = 9) before and after tamoxifen

Animal ID	PR, ms	QRS, ms	QT_C,_ ms	RR, ms	SD nn, ms	RMS SD, ms	TP, ms^2^	VLF, ms^2^	LF, ms^2^	HF, ms^2^	nLF, nu	nHF, nu
Gαs flx cre before tam	32.50 ± 1.50	9.84 ± 1.1	62.28 ± 2.08	96.9 ± 2.46	2.92 ± 0.65	2.08 ± 0.51	131 ± 5.58	60.23 ± 9.2	39.53 ± 7.6	30.21 ± 7.9	55.16 ± 7.9	42.7 ± 7.52
Gαs flx cre after tam	32.60 ± 1.22	9.92 ± 0.87	40.08 ± 2.07*	148.8 ± 4.05*	5.05 ± 1.00	7.14 ± 6.11	160 ± 82	85.12 ± 9.9	36.4 ± 8.9	37.35 ± 6.7	37.08 ± 3.82*	51.0 ± 4.60
Gαs control before tam	34.96 ± 0.82	11.35 ± 0.66	67.75 ± 0.55	95.57 ± 0.064	14.75 ± 3.50	15.20 ± 3.63	297 ± 84	185.35 ± 15.2	42.23 ± 8.1	57.26 ± 12.7	48.34 ± 7.34	51.63 ± 7.33
Gαs control after tam	38.81 ± 0.44	9.93 ± 0.27	67.05 ± 4.46	101.1 ± 0.95	12.51 ± 5.27	7.58 ± 0.62	200 ± 5.72	101.98 ± 5.9	48.6 ± 7.8	45.23 ± 7.2	49.27 ± 3.17	45.61 ± 4.44
Gαi2 flx cre before tam	33.12 ± 0.49	10.41 ± 0.63	69.84 ± 4.98	113.1 ± 8.4	23.8 ± 7.57	11.7 ± 2.66	215 ± 81.4	97.7 ± 8.2	56.1 ± 9.2	49.5 ± 5.8	47.5 ± 6.08	43.35 ± 3.09
Gαi2 flx cre after tam	34.06 ± 0.88	9.96 ± 0.37	69.21 ± 3.43	98.94 ± 7.8*	31.4 ± 6.64	13.6 ± 5.49	1302 ± 432	1201 ± 110	44.6 ± 7.8	31.2 ± 6.7	44.7 ± 5.97	30.21 ± 1.2*
Gαi2 control before tam	35.07 ± 0.31	9.52 ± 0.452	61.63 ± 3.78	119.9 ± 10.1	11.80 ± 4.50	8.24 ± 0.81	134 ± 15.8	65.6 ± 12.7	25.01 ± 5.2	21.76 ± 5.9	36.6 ± 6.15	32.7 ± 2.08
Gαi2 control after tam	36.33 ± 1.04	10.12 ± 0.35	62.36 ± 3.76	123.7 ± 1.7	5.778 ± 5.92	7.12 ± 0.97	248 ± 5.5	171.2 ± 10.2	27.1 ± 3.4	36.2 ± 7.2	35.4 ± 8.31	47.95 ± 3.55

* *P* < 0.05, repeated-measures one-way ANOVA.

The comparisons are between mice before and after tamoxifen (tam) in the various groups over the various parameters. HRV, heart rate variability; RMS, root mean square; TP, total power; VLF, very low frequency; LF, low frequency; HF, high frequency; nLF, normalized low frequency; nHF, normalized high frequency; nu, normalized units.

#### Heart rate variability (HRV) analysis.

**Fig. 2.**
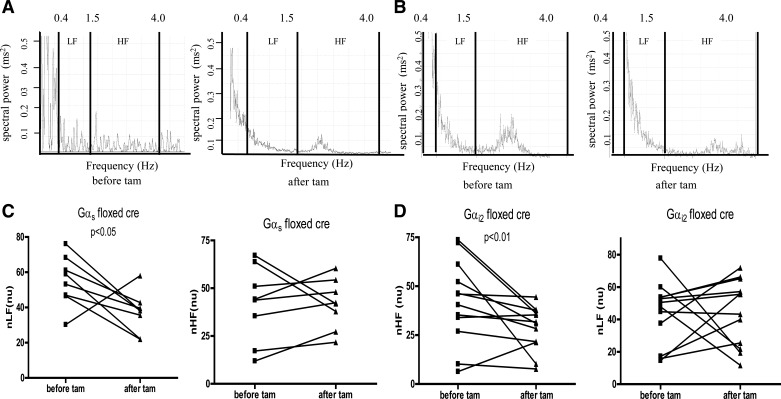
Heart rate variability. Representative power spectrum density traces for Gαs^fl\flx^ cre+ (*A*) and for Gαi2^flx\flx^, cre+ (*B*) before and after tamoxifen administration. Mean normalized LF and HF for Gαs^fl\flx^ cre+ (*C*; *n* = 8) and control (*n* = 8) and for Gαi2^flx\flx^, cre+ (*D*; *n* = 11) and control (*n* = 9) mice.

In both Gαs^flx\flx^ cre+ and Gαi2^flx\flx^ cre+ mice, HRV was altered after tamoxifen treatment. This was detectable in an individual mouse in power spectral density analysis (Fig. 2). Specifically, there was a selective loss of normalized LF power in Gαs^flx\flx^ cre+ mice, while, in contrast, in Gαi2^flx\flx^ cre+ mice, there was a selective loss in normalized HF power (Fig. 2 and Table 1).

#### Pharmacological modulation of heart rate.

**Fig. 3.**
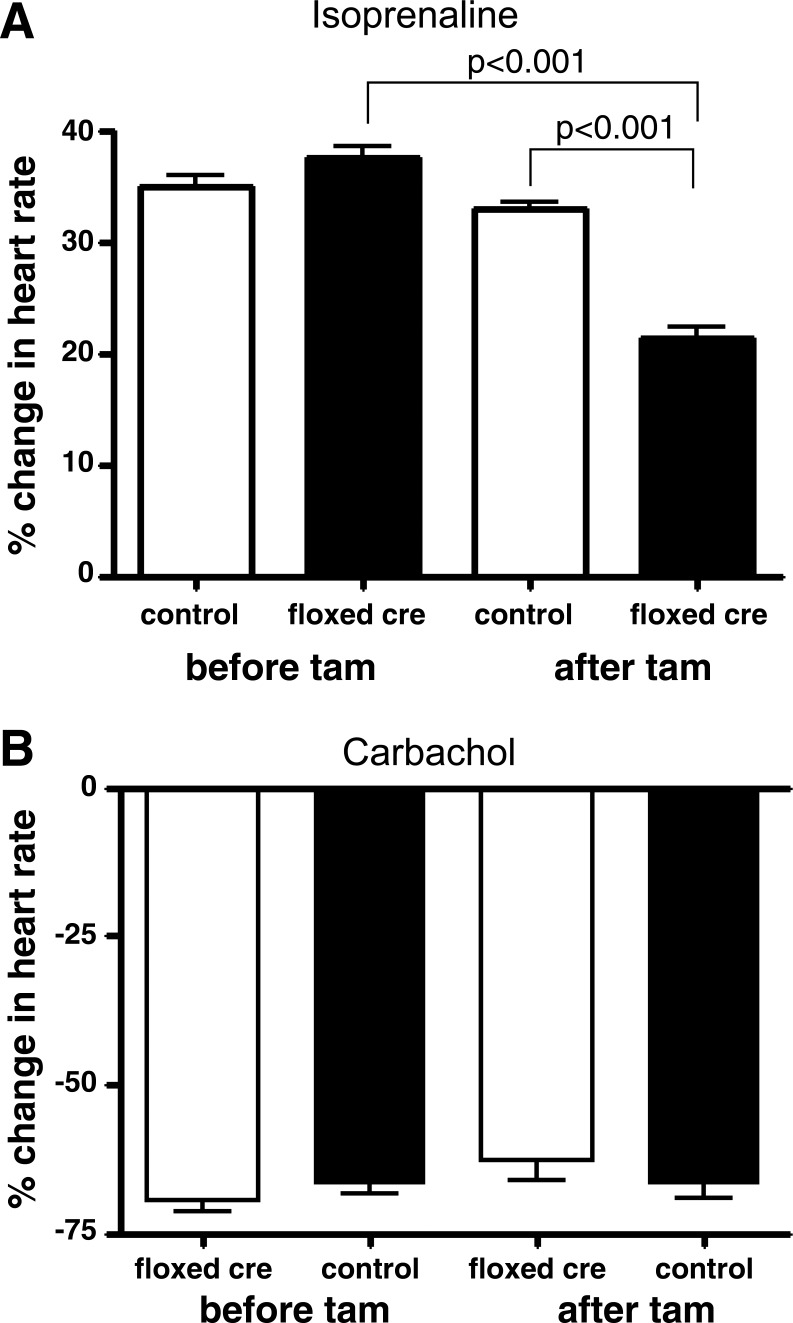
Response of heart rate to pharmacological agents. The percentage change in heart rate in Gαs^fl\flx^ cre+ (*n* = 8) and control (*n* = 8) mice after isoprenaline administration (*top*) and for Gαi2^flx\flx^, cre+ (*n* = 11) and control (*n* = 9) mice after carbachol administration (*bottom*). Values are expressed as means ± SE.

We measured the chronotropic response after the administration of isoprenaline and carbachol in Gαs^flx\flx^ cre+ and Gαi2^flx\flx^ cre+ mice, respectively, after treatment with tamoxifen and compared this to control littermate mice (Fig. 3). The positive chronotropic response to isoprenaline was abrogated but not abolished in Gαs^flx\flx^ cre+ mice, and there was only a small nonsignificant reduction in the negative chronotropic response to carbachol in Gαi2^flx\flx^ cre+ mice. There was no evidence of conduction block or arrhythmias occurring after drug administration.

#### Intrinsic heart rate determination and HRV after atropine and propranolol administration.

**Fig. 4.**
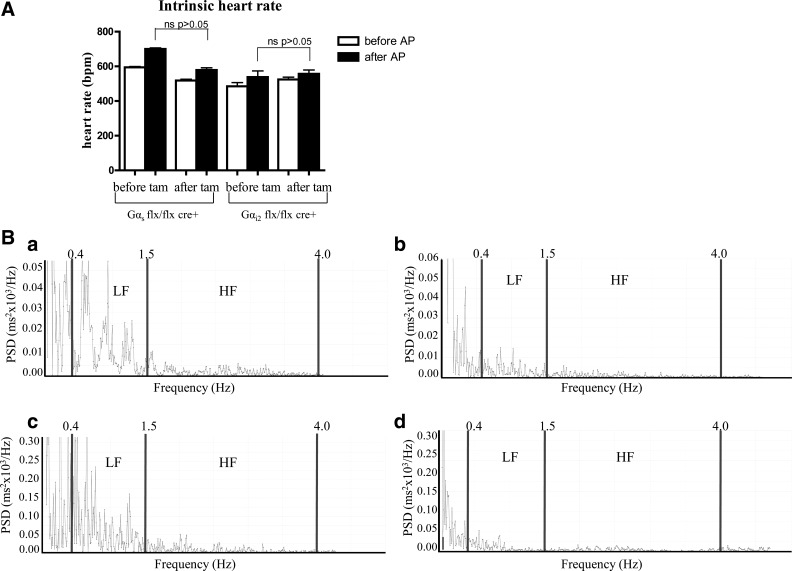
Autonomic blockade. *A*: intrinsic heart rate after autonomic blockade with atropine and propranolol in Gαs^fl\flx^ cre+ and Gαi2^flx\flx^, cre+ mice (*n* = 6 in both groups). Values are expressed as means ± SE. *B*: representative power spectrum density (PSD) traces for Gαs^flx\flx^ cre+ (*a* and *b*) and for Gαi2^flx\flx^, cre+ (*c* and *d*) after prior treatment with tamoxifen and shown are the PSDs before (*left*, *a* and *c*) and after (*right*, *b* and *d*) atropine and propranolol administration.

For complete autonomic blockade, atropine and propanolol were administered together in Gαs^flx\flx^ cre+ and Gαi2^flx\flx^ cre+ mice before and after treatment with tamoxifen, and we compared their intrinsic heart rate and HRV to control littermate mice. In Fig. 4*A* and Table 2, we show that that there was no change in intrinsic heart rate in both Gαs^flx\flx^ cre+ and Gαi2^flx\flx^ cre+. We also examined the effects of the combined atropine and propranolol treatment on HRV before and after tamoxifen treatment in the two groups of mice. We show the data after the administration of tamoxifen in Gαs^flx\flx^ cre+ and Gαi2^flx\flx^ cre+ mice. The administration of the two drugs led to a decrease in HRV across all of the frequency spectra (Table 2). There was no difference between the two genotypes in this behavior before (not shown) or after tamoxifen administration (Fig. 4 and Table 2). In Fig. 4*B*, representative power spectra show abrogation of HF and LF power in both Gαs^flx\flx^ cre+ and Gαi2^flx\flx^ cre+ mice after atropine and propranolol administration (Fig. 4*B*).

**Table 2.** HRV parameters after atropine and propanolol (autonomic blockade) in Gαs^flx^^\flx^ cre and Gαi2^flx\flx^ cre mice

Animal ID	*n*	Mean NN, ms	HR, bpm	SDNN, ms	RMS SD	TP, ms^2^	VLF, ms^2^	LF, ms^2^	LF, nu	HF, ms^2^	HF, nu	LF/HF
Gαi2 before AP	6	107.8 ± 7.1	535 ± 29	8.5 ± 2.9	4.87 ± 0.78	130 ± 23.1	86.2 ± 9.5	32.9 ± 9.7	75.1 ± 9.3	10.2 ± 3.2	23.3 ± 4.7	3.2 ± 0.84
Gαi2 after AP	6	100.3 ± 9.6	597 ± 21	7.4 ± 1.9	3.89 ± 0.28	56.4 ± 10.7**	37.6 ± 5.68*	12.23 ± 5.4**	65.4 ± 10	5.2 ± 1.2*	27.6 ± 6.9	2.2 ± 0.35
Gαs before AP	6	108.2 ± 7.2	555 ± 29	8.8 ± 3.8	5.78 ± 0.34	104 ± 8.9	81.09 ± 10.2	12.8 ± 4.8	55.65 ± 8.2	10.2 ± 2.4	44.3 ± 8.3	1.25 ± 0.32
Gαs after AP	6	98.63 ± 10	606 ± 29	4.9 ± 0.64	3.33 ± 0.87	23.7 ± 9.8**	14.07 ± 6.6*	5.4 ± 1.4*	56.3 ± 9.7	3.1 ± 0.24*	32.5 ± 5.8	1.7 ± 0.34

AP, atropine and propanolol.

** *P* < 0.005,

* *P* < 0.05 before and after administration (repeated-measures one-way ANOVA).

## DISCUSSION

The main findings in this study are that selective deletion of Gαs and Gαi2 in the conduction system of the adult mouse impairs physiological heart rate regulation in the intact awake animal. Mice are nocturnal creatures active at night and more sedentary during the day; thus, they show a characteristic diurnal variation in heart rate. The higher mean rate at night reflects their increased movement and higher sympathetic drive. The selective deletion of Gαs in the conduction system results in loss of normal diurnal variation with the mice no longer tachycardic at night and thus relatively bradycardic compared with control. In contrast, although mice with selective deletion Gαi2 in the conduction system also lose normal diurnal variation, this occurs because they are relatively more tachycardic during the day, consistent with a decline in parasympathetic modulation of the SA node. We also determined the intrinsic heart using pharmacological blockade of the autonomic nervous system with atropine and propranolol. The intrinsic heart rate was not significantly different between the two groups of mice and the deletion of Gαs and Gαi2 in the SA node after tamoxifien administration did not change it. This confirms in vivo that the fundamental inherent pacemaker is not determined by Gαs and Gαi2 and pathways downstream of the G protein.

Heart rate shows physiological variation in rate in a number of frequency domains. In humans, a HF component (0.15 to 0.4 Hz), a LF component (0.04 to 0.15 Hz), and a very low-frequency component (<0.04 Hz) are distinguished, and in the mouse, the frequencies defining each band are 10-fold higher (22a, 24). The high-frequency component is generally thought to reflect vagal input, while the low-frequency component reflects sympathetic and vagal input. However, the latter, in particular, is controversial (22a). Our data support a position broadly in which physiological Gαi2-mediated signaling in the SA node reflects the HF component and Gαs signaling reflects the LF component, and these two are distinct with little crosstalk. In terms of autonomic input, this would correspond to delineation between parasympathetic and sympathetic, accounting largely for most of HF and LF power, respectively. It is worth noting that we use a standard normalization procedure, and once this is done, the correlation between LF and sympathetic activity is clearer (22a). Defining the molecular correlates underlying HRV is not simply of academic interest. HRV is a widely used noninvasive technique to assess autonomic tone, particularly in human studies, and has prognostic significance in patients after myocardial infarction, with hypertension and in many other diseases (22a, 23).

There are some interesting comparisons in HRV data between the mice with conditional genetic deletion of Gαs and Gαi2 in the SA node and those after drug administration and in mice with global genetic deletion of Gαi2. The global genetic deletion of Gαs in mice is embryonically lethal, and thus, the heart rate phenotype of this animal cannot be studied (27). The global genetic deletion of Gαi2 and the administration of atropine, tertiapin, propranolol, and atropine suppressed HRV in the time domain and in the frequency domain and generally had effects across all three frequency spectra (29). In contrast, the effect of conditional deletion in the SA node was more subtle and was revealed when variation in VLF was removed by the normalization of HF and LF. It is worth noting that VLF accounts for the majority of power in the HRV signal. There are some technical caveats that need to be considered. Nonstationarities in the HRV signal can be a confounding factor and, for example, monitoring HRV throughout the day as human subjects go about their daily lives may overestimate the sympathetic contribution (4, 15). We seek to minimize using the recommended approaches, i.e., monitoring the mice at rest at a defined time during the day for a short recording period. Furthermore, it is important that the mice are allowed to recover from surgery (generally 7–10 days) and resume normal activity after probe implantation before measurements are made. These factors are the same between our current and previous studies, and thus, the differences are likely real. Factors likely responsible for the differences are those that lie outside the SA node. For example, effects in the central and peripheral nervous system also have an important influence on HRV. Gαi2 is expressed in the nervous system, and drugs, such as propranolol, with a high lipophilicity cross the blood-brain barrier. In this regard, the HCN4-KiT mouse does not lead to deletion of genes in the brain after the administration of tamoxifen (12).

It is important to clearly define in vivo the molecular isoforms responsible for physiological heart rate regulation. Thus, the *Gnas* gene locus is complex with imprinting (although not in cardiac tissues) and the potential for the generation of a long and extra-long isoforms, whose roles in signaling are not clear (27). The data here implicate canonical Gαs, although the mice do not distinguish long and short forms of Gαs. XLαs, which is a distinct paternal Gαs isoform from the paternal allele, is not affected in these mice. Our results are consistent with recent studies in pigs using adenoviral transfer of small interfering RNA to Gαs (14). Furthermore, there has been debate about the inhibitory G protein isoform underlying physiological heart rate modulation, and we have discussed this in considerable detail elsewhere (1, 29). The fundamental issue is that a wide variety of different approaches and experimental systems have been used. Our data here support an important role for Gαi2 but do not exclude a contribution from other isoforms (see below). We believe that the genetic and in vivo monitoring approach that we have adopted is one of the better combinations to address such integrative physiological questions.

A potential qualification with our data is the response to the pharmacological agents. After selective deletion of Gαs in the conduction system, the response to isoprenaline was significantly attenuated but not abolished, while with Gαi2 deletion, the heart rate-slowing response to carbachol is relatively well preserved. This contrasts with the situation in the global knockout of Gαi2, where it was significantly attenuated. The tests with isoprenaline and carbachol are a pronounced pharmacological challenge in that, for example, with carbachol, the heart rate falls to levels a long way below that normally seen physiologically, even in normal situations, such as sleep. It is known that the sinoatrial node is actually quite an extensive and heterogenous structure and that the primary pacemaking site moves from one region to another, as the sympathovagal balance changes (17, 21). Thus, it is possible that the pacemaking site shifts to a small region that is not solely dependent on the deleted G protein or a site in which the HCN4 promoter driven cre-mediated deletion is not so complete. A further consideration is that the dose of isoprenaline, while leading to a close to maximal heart rate response, is considered to be relatively low (20). It is possible that the variances in behavior between carbachol and isoprenaline are accounted for by differences in the relative position of the single dose on the dose-response curve.

There is great interest in the role that heart rate per se has in the outcome of heart failure after the use of ivabridine in the SHIFT trial (7). These mouse lines may be useful resources in addressing this interesting question, as they have changes in heart rate generated independently of pharmacological manipulation. While the mouse is valuable for understanding the molecular basis of cardiac function, especially given the relative ease and sophistication of the manipulation of the genome, it has limitations. The mouse heart rate is ∼10-fold higher than that in a human, and there are differences in ion channel expression in various cardiac cells across the species. In addition, rat and mouse show a negative force-frequency relationship, while it has a positive slope in larger mammals, such as rabbits (16). However, it is clear that in the range of normal physiological heart rates, Gαs and Gαi2 in the sinoatrial node have an important role in determining normal heart rate and normal physiological variability.

## DISCLOSURES

No conflicts of interest, financial or otherwise, are declared by the authors.

## AUTHOR CONTRIBUTIONS

Author contributions: S.S., R.A., L.S.W., A.L., L.B., and A.T. conception and design of research; S.S., R.A., J.A., and M.C. performed experiments; S.S. and R.A. analyzed data; S.S., R.A., and A.T. interpreted results of experiments; S.S. and R.A. prepared figures; S.S., R.A., and A.T. drafted manuscript; S.S., R.A., L.S.W., A.L., L.B., and A.T. edited and revised manuscript; S.S., R.A., J.A., L.S.W., M.C., A.L., L.B., and A.T. approved final version of manuscript.

## References

[1] Ang R Opel A Tinker A The role of inhibitory G proteins and regulators of G protein signaling in the in vivo control of heart rate and predisposition to cardiac arrhythmias. Front Physiol 3: 96, 2012 22783193 10.3389/fphys.2012.00096 PMC3390690

[3] Baruscotti M Bucchi A Viscomi C Mandelli G Consalez G Gnecchi-Rusconi T Montano N Casali KR Micheloni S Barbuti A DiFrancesco D Deep bradycardia and heart block caused by inducible cardiac-specific knockout of the pacemaker channel gene Hcn4. Proc Natl Acad Sci USA 108: 1705–1710, 2011 21220308 10.1073/pnas.1010122108 PMC3029742

[4] Berntson GG Bigger JT Jr Eckberg DL Grossman P Kaufmann PG Malik M Nagaraja HN Porges SW Saul JP Stone PH van der Molen MW Heart rate variability: origins, methods, and interpretive caveats. Psychophysiology 34: 623–648, 1997 9401419 10.1111/j.1469-8986.1997.tb02140.x

[5] Birnbaumer L Expansion of signal transduction by G proteins. The second 15 years or so: from 3 to 16 alpha subunits plus betagamma dimers. Biochim Biophys Acta 1768: 772–793, 2007 17258171 10.1016/j.bbamem.2006.12.002 PMC1993906

[6] Bogdanov KY Maltsev VA Vinogradova TM Lyashkov AE Spurgeon HA Stern MD Lakatta EG Membrane potential fluctuations resulting from submembrane Ca^2+^ releases in rabbit sinoatrial nodal cells impart an exponential phase to the late diastolic depolarization that controls their chronotropic state. Circ Res 99: 979–987, 2006 17008599 10.1161/01.RES.0000247933.66532.0b

[7] Bohm M Swedberg K Komajda M Borer JS Ford I Dubost-Brama A Lerebours G Tavazzi L Heart rate as a risk factor in chronic heart failure (SHIFT): the association between heart rate and outcomes in a randomised placebo-controlled trial. Lancet 376: 886–894, 2010 20801495 10.1016/S0140-6736(10)61259-7

[8] Chen M Gavrilova O Zhao WQ Nguyen A Lorenzo J Shen L Nackers L Pack S Jou W Weinstein LS Increased glucose tolerance and reduced adiposity in the absence of fasting hypoglycemia in mice with liver-specific Gs alpha deficiency. J Clin Invest 115: 3217–3227, 2005 16239968 10.1172/JCI24196 PMC1257533

[9] Ecker PM Lin CC Powers J Kobilka BK Dubin AM Bernstein D Effect of targeted deletions of β1- and β2-adrenergic-receptor subtypes on heart rate variability. Am J Physiol Heart Circ Physiol 290: H192–H199, 2006 16113068 10.1152/ajpheart.00032.2005

[10] Gehrmann J Hammer PE Maguire CT Wakimoto H Triedman JK Berul CI Phenotypic screening for heart rate variability in the mouse. Am J Physiol Heart Circ Physiol 279: H733–H740, 2000 10924073 10.1152/ajpheart.2000.279.2.H733

[11] Herrmann S Stieber J Stockl G Hofmann F Ludwig A HCN4 provides a ‘depolarization reserve’ and is not required for heart rate acceleration in mice. EMBO J 26: 4423–4432, 2007 17914461 10.1038/sj.emboj.7601868 PMC2063478

[12] Hoesl E Stieber J Herrmann S Feil S Tybl E Hofmann F Feil R Ludwig A Tamoxifen-inducible gene deletion in the cardiac conduction system. J Mol Cell Cardiol 45: 62–69, 2008 18538341 10.1016/j.yjmcc.2008.04.008

[13] Lakatta EG DiFrancesco D What keeps us ticking: a funny current, a calcium clock, or both? J Mol Cell Cardiol 47: 157–170, 2009 19361514 10.1016/j.yjmcc.2009.03.022 PMC4554526

[14] Lugenbiel P Bauer A Kelemen K Schweizer PA Becker R Katus HA Thomas D Biological heart rate reduction through genetic suppression of Galpha(s) protein in the sinoatrial node. J Am Heart Assoc 1: 2012 10.1161/JAHA.111.000372 PMC3487376 23130123

[15] Magagnin V Bassani T Bari V Turiel M Maestri R Pinna GD Porta A Non-stationarities significantly distort short-term spectral, symbolic and entropy heart rate variability indices. Physiol Meas 32: 1775–1786, 2011 22027399 10.1088/0967-3334/32/11/S05

[16] Maier LS Bers DM Pieske B Differences in Ca^2+^-handling and sarcoplasmic reticulum Ca^2+^-content in isolated rat and rabbit myocardium. J Mol Cell Cardiol 32: 2249–2258, 2000 11113000 10.1006/jmcc.2000.1252

[17] Monfredi O Dobrzynski H Mondal T Boyett MR Morris GM The anatomy and physiology of the sinoatrial node—a contemporary review. Pacing Clin Electrophysiol 33: 1392–1406, 2010 20946278 10.1111/j.1540-8159.2010.02838.x

[18] Pumprla J Howorka K Groves D Chester M Nolan J Functional assessment of heart rate variability: physiological basis and practical applications. Int J Cardiol 84: 1–14, 2002 12104056 10.1016/s0167-5273(02)00057-8

[19] Schmittgen TD Livak KJ Analyzing real-time PCR data by the comparative C(T) method. Nat Protoc 3: 1101–1108, 2008 18546601 10.1038/nprot.2008.73

[20] Shan J Kushnir A Betzenhauser MJ Reiken S Li J Lehnart SE Lindegger N Mongillo M Mohler PJ Marks AR Phosphorylation of the ryanodine receptor mediates the cardiac fight or flight response in mice. J Clin Invest 120: 4388–4398, 2010 21099118 10.1172/JCI32726 PMC2993575

[21] Shibata N Inada S Mitsui K Honjo H Yamamoto M Niwa R Boyett MR Kodama I Pacemaker shift in the rabbit sinoatrial node in response to vagal nerve stimulation. Exp Physiol 86: 177–184, 2001 11429632 10.1113/eph8602100

[22] Stauss HM Heart rate variability. Am J Physiol Regul Integr Comp Physiol 285: R927–R931, 2003 14557228 10.1152/ajpregu.00452.2003

[22a] **Task Force of the European Society of Cardiology and the North American Society of Pacing and Electrophysiology. **Heart rate variability. Standards of measurement, physiological interpretation, and clinical use. Eur Heart J 17: 354–381, 1996 8737210

[23] Thayer JF Yamamoto SS Brosschot JF The relationship of autonomic imbalance, heart rate variability and cardiovascular disease risk factors. Int J Cardiol 141: 122–131, 2010 19910061 10.1016/j.ijcard.2009.09.543

[24] Thireau J Zhang BL Poisson D Babuty D Heart rate variability in mice: a theoretical and practical guide. Exp Physiol 93: 83–94, 2008 17911354 10.1113/expphysiol.2007.040733

[25] Uechi M Asai K Osaka M Smith A Sato N Wagner TE Ishikawa Y Hayakawa H Vatner DE Shannon RP Homcy CJ Vatner SF Depressed heart rate variability and arterial baroreflex in conscious transgenic mice with overexpression of cardiac Gsα. Circ Res 82: 416–423, 1998 9506701 10.1161/01.res.82.4.416

[26] Ustyugova IV Zhi L Abramowitz J Birnbaumer L Wu MX IEX-1 deficiency protects against colonic cancer. Mol Cancer Res 10: 760–767, 2012 22550081 10.1158/1541-7786.MCR-11-0556 PMC3465947

[27] Weinstein LS Xie T Zhang QH Chen M Studies of the regulation and function of the Gs alpha gene Gnas using gene targeting technology. Pharmacol Ther 115: 271–291, 2007 17588669 10.1016/j.pharmthera.2007.03.013 PMC2031856

[28] Wickman K Nemec J Gendler SJ Clapham DE Abnormal heart rate regulation in GIRK4 knockout mice. Neuron 20: 103–114, 1998 9459446 10.1016/s0896-6273(00)80438-9

[29] Zuberi Z Birnbaumer L Tinker A The role of inhibitory heterotrimeric G-proteins in the control of in-vivo heart rate dynamics. Am J Physiol Regul Integr Comp Physiol 295: R1822–R1830, 2008 18832081 10.1152/ajpregu.90625.2008 PMC3395347

